# The Evolution and the Impact of Refractive Errors on Academic Performance: A Pilot Study of Portuguese School-Aged Children

**DOI:** 10.3390/children9060840

**Published:** 2022-06-06

**Authors:** Clara Martinez-Perez, Cristina Alvarez-Peregrina, Rita Brito, Miguel Ángel Sánchez-Tena, Grupo de Investigação Optovisão ISEC Lisboa

**Affiliations:** 1 ISEC LISBOA—Instituto Superior de Educação e Ciências, 1750-179 Lisbon, Portugal; rita.brito@iseclisboa.pt (R.B.); masancheztena@ucm.es (M.Á.S.-T.); 2 Department of Optometry and Vision, Faculty of Optics and Optometry, Universidad Complutense de Madrid, 28037 Madrid, Spain; cristina_alvarez@ucm.es; 3 The Grupo de Investigação Optovisão ISEC Lisboa, 1750-179 Lisbon, Portugal; henrique.nascimento@iseclisboa.pt

**Keywords:** myopia, vision, academic performance

## Abstract

The relationship between vision and academic performance has been discussed for a long time, with special emphasis on visual factors associated with learning problems. The objective of this pilot study is to obtain an initial idea about the evolution and the impact of refractive errors on school-aged children. A visual examination was performed on 252 children between the ages of 6 and 11 years, which consisted of objective refraction, subjective refraction, and accommodative and binocular tests. No significant differences were observed regarding the refractive state when taking academic performance into account (*p* > 0.05). However, it was determined that academic performance was better among children with a negative spherical equivalent. Studies with a larger sample size must be conducted to verify the results that were attained in this present pilot study, and these must likewise look at possible ways in which strategies can be implemented in schools to reduce myopia progression.

## 1. Introduction

Vision plays an essential role in the acquisition of skills during childhood, which includes language, hand-eye coordination, and the interpretation of facial expressions [1]. Vision is a key component of a child’s learning and educational progress, therefore visual impairment could result in a reduced motivation to explore their surroundings, initiate social interactions, or manipulate objects. Students with visual impairment are often unable to share experiences with their peers, which has an impact on their development of social skills [2]. It is estimated that 75% of learning comes from vision, and one in five children presents visual alterations that could be corrected. If visual alterations are not corrected or detected in time, they can result in a child’s educational failure [3].

Uncorrected refractive errors are the leading cause of visual impairment worldwide [4], and they affect approximately 12.8 million children from 5 to 15 years of age [5]. Many population-based studies have analyzed the prevalence of myopia among children in different countries. According to these studies, Taiwan has the highest myopia prevalence in school-aged children, with a rate of 36.4% [6] in 8-year-old children, followed by Singapore with a rate of 34.7% [6], Shanghai with a rate of 30.8% [7], and Malaysia [8] with a rate of 14.0%. In non-oriental Asian countries, an increased prevalence of myopia has also been reported, although this rate is lower than in Asia.

Although there are fewer studies in European countries, differences between the countries have been detected. In England, the Aston Eye Study reported a prevalence of 9.4% in children aged between 6 and 7 years old and 29.4% in children aged between 12 and 13. However, Northern Ireland reported a prevalence rate of 2.8% and 17.7%, respectively, in children of the same age [9,10]. The Netherlands reported a lower prevalence of myopia—approximately 2.4% in a sample of 5711 6-year-old children [11]. In Poland, the myopia prevalence was 2.0% in 6-year-old children, 8.4% in 8-year-old children, and 14.7% in 12-year-old children [12]. As shown in our results, Portugal had a similar prevalence rate; however, Spain had a higher prevalence rate of 20.1% in children aged between 5 and 7 years old [13].

Around 40% of school-aged children experience visual problems, which may harm their visual function [14]. Scientific evidence has shown that uncorrected or poorly corrected refractive errors can affect a child’s academic development [15,16,17,18,19,20]. Nevertheless, not every visual problem results in reduced visual acuity. For example, hyperopia does not reduce visual acuity until 2 dioptres. However, this also means that hyperopia can go unnoticed in visual screening tests that are performed to measure high contrast visual acuity and binocular vision [21,22]. As a result, the academic performance of children with hyperopia could be affected. There is also strong evidence to suggest that academic performance and educational success have a long-term effect on social, economic, and health results [23,24].

These findings were recently questioned in a study conducted by Hopkins et al. [25], which demonstrated that most of the studies use suboptimal research methods, small sample sizes, and poorly-designed trials. As a result, such studies are less capable of determining association or causality.

Most educational activities, such as reading and writing, are performed at a near distance for an extended period [26]. Moreover, with the increasing use of electronic devices, such as smartphones, tablets, and e-books, the use of screens at a near distance and for an extended period has become more relevant. Likewise, screens are widely used for educational and for leisure purposes [27]. Given that uncorrected hyperopia is the refractive error with the most significant impact on near vision, its impact on learning may further increase [28,29].

Although uncorrected hyperopic eyes may be able to compensate for the refractive error through temporary additional accommodation, the demand for continuous additional accommodation may result in asthenopia and headaches, which could result in children unconsciously avoiding near-vision activities due to visual discomfort [30]. As a result, children with hyperopia may not be aware of their “atypical” vision, or they may not be able to explain what it is that they are experiencing. A recent study showed that hyperopia correction improved accommodative performance for prolonged reading tasks in the majority of participants [31].

Likewise, there are other determinants for the development of myopia, such as prolonged near-vision tasks, non-corrected refractive errors, bad posture, bad reading habits, inadequate gaze breaks, a lack of outdoor activity, and excessive use of television or digital devices [32]. Previous studies on refractive errors also recommended the use of good lighting and ensuring an easy and natural posture while reading [33].

For over a century, myopia has been associated with the highest levels of learning achievements [34,35], nonetheless, further scientific evidence is required to fully understand the causative connection [36]. There is a hereditary component in both myopia and level of education; however, genetics cannot explain the rapid increase in the prevalence of myopia in one or two generations [37,38]. Randomized and controlled clinical trials have demonstrated that spending time outdoors during childhood can provide partial protection against myopia onset [39,40]. On the contrary, the association between myopia and the time on near-vision tasks, such as reading, is not stable in all studies [41,42]. Moreover, the time children spend outdoors tends to be separate from their near-vision activities as, in general, both measurements are not correlated [43,44].

Therefore, it is unclear if: (1) a higher educational level causes myopia, (2) children with myopia spend more time doing near-vision tasks, therefore meaning that they attain better educational results, (3) children with myopia are more intelligent, or (4) socioeconomic position leads to more years being spent in education and a higher incidence of myopia [45]. The reason for this is that randomized clinical trials that limit children’s education would not be ethical.

As a result, there is a lack of research regarding the impact of vision on academic achievement. Therefore, this pilot study aims to obtain an initial idea about the evolution and the impact of refractive errors on school-aged children.

## 2. Materials and Methods

An observational, prospective, cross-sectional, and multi-center study was carried out. This study was conducted in five schools in Lisbon, from October to December 2021. A total of 252 children, aged between 6 to 12 years of age participated in the study.

With regard to ethical issues, the children’s parents signed the informed consent form, stating that they had clearly understood the study’s objectives. This research followed the principles of the Declaration of Helsinki and it was approved by the Ethics Committee of the Superior Institute of Education and Sciences (ISEC Lisbon) on 5 November 2021 under code CE/2022/03/01.

All of the children underwent an optometry test, which consisted of:

(1) visual acuity: best-corrected and non-corrected;
(2) objective refraction: non cycloplegic retinoscopy;
(3) subjective refraction;
(4) binocular vision and accommodative tests: cover-uncover, alternate cover, ocular motility, accommodative delay, Worth’s test, near point of convergence, stereopsis, and color vision.

Moreover, the schools were asked about the average grades obtained in science and humanities subjects in the previous academic year.

The statistical analysis was performed using the SPSS 25.0 computer program (SPSS Inc., Chicago, IL, USA). The normal distribution of variables was conducted using the Kolmogorov–Smirnov test with a significance level of 0.05. As a result of a non-parametric distribution, the following tests were used: the non-parametric Kruskal–Wallis or U Mann–Whitney test for quantitative variables and the Chi-square test for qualitative variables. Passing–Bablok linear regression was employed to analyze the association between the continuous quantitative variables. Quantitative variables were described using the mean ± standard deviation (SD) or the median and the interquartile range, depending on the distribution.

The spherical equivalent (SE) was calculated based on the objective refraction as the value of the sphere plus half of the cylinder. With regard to the classification of refractive error, hypermetropia was considered as D ≥ +0.5 D, myopia as D ≤ −0.5 D, and emmetropia as D values between −0.5 D and +0.5 D [25].

## 3. Results

With regard to gender, 49.6% of participants were female (*n* = 125) and 50.4% were male (*n* = 127). The average age of the participants was 8.0 ± 1.5 years, and the median age was eight years old, with an interquartile range of two.

### 3.1. Prevalence of Refractive Errors

The prevalence of myopia was 9.5% (*n* = 22), emmetropia was 34.1% (*n* = 86), and hyperopia was 56.3% (*n* = 144). There were no statistically significant differences recorded in terms of gender and refractive errors (*p* > 0.05).

**Figure 1:**
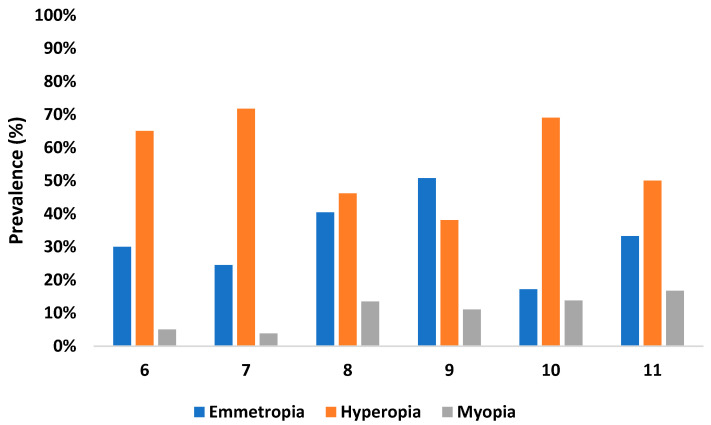
Prevalence of refractive errors regarding age.

Figure 1 shows that there was a gradual decrease in the rate of hyperopia and a gradual increase in the rate of myopia among older subjects (*p* = 0.010). Likewise, between 6 and 11 years of age, the rate of hyperopia decreased by 25.3% (23.2% at 6 years of age and 2.1% at 11). The rate of myopia also increased by 12.5% (4.2% at 6 years of age and 16.7% at 11).

Of all participants with myopia, 81% (*n* = 17) presented low myopia (−0.50 < D > −3) and 19% (*n* = 5) presented moderate myopia (−3.00 D < D > −6.00 D).

**Figure 2:**
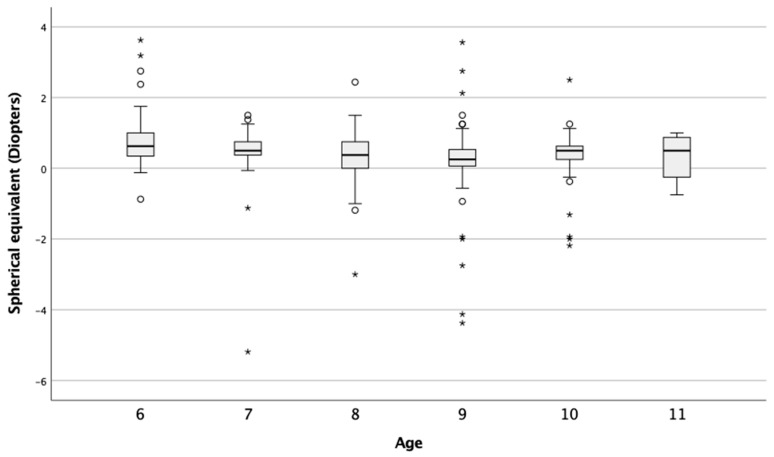
Value of the spherical equivalent according to age. Box = 1 SD, line = median, whiskers = confidence interval of 95%, o = extreme value, * = outliers.

The mean value of the spherical equivalent among all participants was 0.39 ± 0.97, with a range from −5.19 D to 3.63 D. With regard to gender, the mean value of the spherical equivalent was 0.40 ± 1.03 (range: −4.37 D to 3.56 D) in female participants and 0.38 ± 0.92 (range: from −5.19 D to 3.63 D) in male participants. With regard to age, Figure 2 shows that there was a clear tendency of developing myopia as the children’s ages increased (*p* = 0.033). The mean value for 6-year-old children was 0.77 ± 0.82 (range: from −0.87 D to 3.63 D) and for 11-year-old children 0.31 ± 0.68 (range: from −0.75 D to 1.00 D).

Table 1 shows the evolution of the refractive state, taking into account the child’s school year. Between the first and the sixth year of school, the rate of myopia increased from 4.3% to 14.3% (*p* = 0.040).

### 3.2. Academic Performance

Academic performance decreased (*p* = 0.022) in the higher school years. However, no significant differences were observed regarding the refractive state when taking academic performance into account (*p* > 0.05). Table 2 shows no correlation between the SE value and academic performance across the school years (*p* > 0.05). Nonetheless, when the Passing–Bablok regression was applied, it was observed that the higher the academic performance, the more positive the SE for the first (β = 0.013, *p* = 0.003) and the third (β = 0.055, *p* < 0.001) year students. To the contrary, the SE was more negative when academic performance was higher among second (β = −0.087, *p* < 0.001) and fourth (β = −0.036, *p* = 0.002) year students. There was no correlation between academic performance and the refractive state among fifth-year students, (*p* > 0.05).

After analyzing the students from all the school years, it was determined that academic performance was better among children with a negative SE (β = −0.014, *p* < 0.001).

No correlation was found between binocular state and academic performance (*p* > 0.005). Neither have significant differences been found between gender and academic performance (*p* = 0.316).

## 4. Discussion

This study is the first to analyze how refractive errors affect children of school age in the Portuguese region of Lisbon. There has been an increased prevalence of myopia worldwide in recent years. As shown in our results, Portugal had a similar prevalence rate to that of England [9] and Poland [12]; however, Spain had a higher prevalence rate of 20.1% in children aged between 5 and 7 years old [13]. There are two possible reasons for this. The first is that, unlike the present study, most of the samples obtained in the Spanish study were from urban areas, and, as shown in previous studies, the living environment can influence the onset and progression of myopia. Population-based studies have shown that there is a higher prevalence of myopia among children living in urban areas than among those living in rural areas [47]. Another possible reason why the results differed could be the lower number of subjects, given that this study only covered the region of Lisbon. Future studies must be comprised of the same number of participants as those studies conducted in Spain to confirm the differences between the border countries. It is worth mentioning that a study by Queirós et al. in 2009 found that the prevalence rate for myopia in northern Portugal was 29.8% for an average age between 40.08 ± 18.75 [48]; 31.1% of the participants aged between 4 and 19 years were myopes. This, once again, suggests that myopia prevalence can vary depending on the studied area, therefore confirming the need for a multicentric study to be conducted covering the different Portuguese regions.

Given that the school years agree with the age at which myopia usually develops and progresses, it has been suggested that it could be related to higher levels of education. Besides this study, this fact has also been proven in numerous studies involving a huge variety of populations [49,50,51]. In Asian and South Asian countries, there was a high prevalence of myopia due to their demanding educational requirements and the intense educational system [52,53]. In Europe, an increased level of education has been related to increased levels of myopia [50]. The incidence of myopia among orthodox Jewish boys who spend a great deal of time reading religious texts at a near distance was higher than the incidence of myopia among girls in Jerusalem [54]. Thus, exposure to an intensive educational system at an early age is a potential risk factor [55,56].

In turn, a higher educational level has also been related to an increased axial length. In China, the AL increased by 0.60 mm for every ten years of studying [56]. The randomized mendelian analysis demonstrated that for every additional year of study, the myopic SE rises by −0.27 D [57]. In contrast, this study did not find any association between the spherical equivalent and academic achievement. This may be due to the low myopic rate that was observed. Nevertheless, as in the studies by Shneor et al. [58] or Mirshani et al. [51], the spherical equivalent tends to be more negative among students in later school years. Therefore, despite no relationship with our sample size being found, the fact that the spherical equivalent becomes more negative suggests that it is likely that there could be significant differences with a larger sample size and a wider age range.

It is worth mentioning that in the study carried out by Jorge et al. [59] in 2007 on a group of Portuguese undergraduate university students, the prevalence of myopia increased more rapidly in the early years. The results of the study by Yang et al. [60] on the impact of refractive status on academic performance were similar to those attained in this study. Therefore, it has been determined that myopic children perform better than hyperopic and emmetropic children. The exact mechanism which links the increased incidence of myopia with an increased level of education remains unknown. Although increased time spent doing near-vision activities has been suggested, nonetheless, its effects have been disputed in several studies [61,62,63].

In this study, no relationship was found between binocular status and academic performance. However, these results differ from those reported in the study by Álvarez-Peregrina et al. [64] in which students who presented with binocular alterations attained a poorer academic performance. The study by Lança et al. [65] presented similar results given that children with strabismus presented lower visual acuity and their academic performance was affected.

With regards to the limitations of this study, it is worth noting that a low sample size can sometimes cause false negatives or false positives when analyzing the association. In turn, the academic variable could be not well selected because it is the previous year’s grade; and, moreover, grades obtained in science and humanities subjects could be not representative of the learning capacities of the children. This study provided preliminary insight into how refractive status varies as the level of education increases. However, as mentioned in the study carried out by Hopkins et al. [16], well-designed experimental studies with an adequate sample size must be conducted to attain a better understanding of how refractive status may be affected. Currently, the veracity of many published studies is affected by methodological limitations.

Therefore, the results obtained in this study are useful for future research conducted to analyze how refractive status varies with educational level as well as looking at possible ways of slowing this progression.

## 5. Conclusions

This study confirms that academic performance decreases with increasing school age. There are no significant differences between refractive status and academic performance. However, the study confirms how the spherical equivalent becomes more negative in the latest school years. Although, no significant differences were found between academic performance and refractive error or binocular vision.

One of the limitations of this study is the low sample size and the fact that the grades of the last course have been taken. Therefore, studies with a larger sample size must be conducted to verify the results that were attained in this present pilot study, and, likewise, these must look at possible ways in which strategies can be implemented in schools to reduce myopia progression.

In addition, it is recommended that children have annual visual examinations in order to prevent poor academic performance and an increase in the prevalence of myopia.

## Figures and Tables

**Table 1:** Demographic data and the refractive state of the study group.

Characteristics	School Year						Total	*p-*Value
	1	2	3	4	5	6		
**No. of participants**	47	50	54	69	25	7	252	-
**Age**								-
Mean ± SD	5.9 ± 0.4	7.0 ± 0.4	7.9 ± 0.4	9.0 ± 0.4	10.0 ± 0.5	10.7 ± 0.5	8.0 ± 1.5	
Median [IQR]	6 [2]	7 [3]	8 [2]	9 [2]	10 [3]	11 [46]	8 [6]	
**Spherical equivalent (D)**	0.56 ± 1.56	0.42 ± 0.92	0.35 ± 0.78	0.20 ± 1.18	0.16 ± 1.12	0.45 ± 0.57	0.35 ± 1.12	-
Mean ± DE								
**Hyperopia**	32 (68.1)	34 (68.0)	26 (48.1)	30 (43.5)	15 (60.0)	5 (71.4)	142 (56.3)	0.040
n (%)								
**Myopia**	2 (4.3)	2 (4.0)	7 (13.0)	8 (11.6)	4 (16.0)	1 (14.3)	24 (9.5)	
n (%)								

**Table 2:** Academic performance according to the spherical equivalent.

Degree	*n*	Spearman Correlation			Passing-Bablok Regression			
		Total ρ (*p*-Value)	Science ρ (*p*-Value)	Humanities ρ (*p*-Value)		Total	Science	Humanities
**1**	47	0.172 (0.249)	−0.306 (0.036)	0.086 (0.565)	**β**	0.013	−0.141	0.007
					**(*p*-value)**	(0.003)	(<0.001)	(0.002)
					**Intercept**	−3.00	−2.21	−3.00
					**(95% CI)**	(−3.00 to −3.00)	(−3.00 to −0.8)	(−3.00 to −3.00)
					**Slope**	1.00	0.82	1.00
					**(95% CI)**	(1.00 to 1.00)	(0.37 to 1.00)	(1.00 to 1.00)
**2**	50	−0.113 (0.436)	−0.204 (0.155)	−0.114 (0.429)	**β**	−0.087	−0.166	−0.114
					**(*p*-value)**	(<0.001)	(<0.001)	(<0.001)
					**Intercept**	−1.25	−0.40	−1.25
					**(95% CI)**	(−4.00 to −0.31)	(−4.01 to 0.14)	(−4.00 to −0.31)
					**Slope**	0.50	0.265	0.5
					**(95% CI)**	(0.25 to 1.00)	(0.12 to 1.38)	(0.25 to 1.00)
**3**	54	−0.077 (0.579)	−0.103 (0.459)	−0.060 (0.667)	**β**	0.055	−0.125	0.017
					**(*p*-value)**	(<0.001)	(<0.001)	(<0.001)
					**Intercept**	−4.00	−4.00	4.00
					**(95% CI)**	(−4 to 3.77)	(−4 to 3.77)	(3.20 to 4.00)
					**Slope**	1.00	1.00	0
					**(95% CI)**	(1.12 to 1.00)	(1.13 to 1.00)	(0.00 to 1.23)
**4**	69	−0.014 (0.909)	0.139 (0.256)	−0.001 (0.993)	**β**	−0.036	0.053	−0.042
					**(*p*-value)**	(0.002)	(<0.001)	(<0.001)
					**Intercept**	−2.33	−4.57	−5.22
					**(95% CI)**	(−6.22 to −1.25)	(−3.00 a −1.70)	(−3.00 to −1.7)
					**Slope**	0.8	0.63	1.75
					**(95% CI)**	(0.5 to 2.02)	(0.63 a 1.00)	(0.63 to 1.00)
**5**	25	−0.028 (0.898)	−0.137 (0.523)	0.203(0.342)	**β**	−0.030	−0.063	0.137
					**(*p*-value)**	(0.620)	(0.139)	(0.512)
					**Intercept**	−2.37	−9.25	−5.50
					**(95% CI)**	(−3.00 to −0.28)	(−4.00 to −0.50)	(−3.00 to 1.75)
					**Slope**	1	2.5	2
					**(95% CI)**	(0.26 to 1.00)	(0.25 to 1.00)	(0.75 to 1.00)
**6**	7	NA	NA	NA		NA	NA	NA

NA: Not available (There is no adequate sample size for the statistical analysis).

## Data Availability

Not applicable.
